# Sexual Boundary Violations via Digital Media Among Students

**DOI:** 10.3389/fpsyg.2021.755752

**Published:** 2022-01-05

**Authors:** Juergen Budde, Christina Witz, Maika Böhm

**Affiliations:** ^1^University of Flensburg, Flensburg, Germany; ^2^Merseburg University of Applied Sciences, Merseburg, Germany

**Keywords:** sexting, sexual boundary violations, group discussion, gender, adolescents, sexual identity, sexual socialization

## Abstract

As digital media becomes more central to the lives of adolescents, it also becomes increasingly relevant for their sexual communication. Sexting as an important image-based digital medium provides opportunities for self-determined digital communication, but also carries specific risks for boundary violations. Accordingly, sexting is understood either as an everyday, or as risky and deviant behavior among adolescents. In the affectedness of boundary violations gender plays an important role. However, it is still unclear to what extent digital sexual communication restores stereotypical gender roles and restrictive sexuality norms or, alternatively, enables new spaces of possibility. In this sense, current research points to a desideratum regarding adolescents’ orientations toward sexting as a practice between spaces of possibility and boundary violations. This paper discusses the possibilities, but also the risks, of intimate digital communication among adolescents. The main question is, how adolescents themselves perceive sexting practices and how they position themselves between both spaces for possibility and for the exchange of unwanted sexual content. For this purpose, orientations toward normalities and gender of students are reconstructed. To answer these questions, twelve single-sex, group discussions were carried out with students aged 16 and 17 at five different secondary schools in northern Germany. A total of 20 boys and 22 girls took part. The group discussions were structured by a narrative generating guideline. The analysis draws its methodology from the Documentary Method, regarding implicit and explicit forms of knowledge and discourse. It results in a typology of three types with different orientations. The study shows, that most of the students consider sexting to be a risky practice; only one type shows normality in the use of sexting. At the same time, some of the young people are interested in experimenting with image-based intimate digital communication. Further, gender differences in use and affectedness are also documented. In this way, orientations toward gender stereotypes “favor” both the attribution of responsibility to girls, and overlook the responsibility of students who perpetrated the boundary violation. The orientations of adolescents should be taken more into account in research as well as in educational programs for the prevention of sexual violence.

## Introduction

Sexuality is a culturally and historically mutable concept that has transformed markedly over the past 100 years – and continues to do so. Representations of sexuality, norms, values, and practices are adaptable, and are closely connected to specific historical and cultural contexts. From early childhood onward, the process of sexual socialization allows individual attitudes, positions and structures of desire to form in engagement with social sexual norms and values. These individual sexual “scripts” both describe sexual identity and shape individual sexual experiences and actions. Especially during adolescence, the formation of sexual identity is characterized by specific dynamics.

In recent decades, the environments of children, adolescents and adults have rapidly digitalized. In particular for adolescents, digital media represent an important part of their lived reality, and are also used to shape sexual activity and communication online (Wachs et al., 2021). Correspondingly, the significance of digital media in the process of sexual socialization for adolescents has increased. The range of media on offer means that instances and forms of sexual communication and interactions during adolescence are becoming more varied, shaping sexual socialization in the process (Murray and Crofts, 2015). This allows further spaces for the development of a self-determined sexuality to emerge; the use of such spaces, however, brings with it multiple risks for sexual self-determination due to boundary violations, misconduct, and victimization.

One particular phenomenon that has gained increasing attention in public, educational science and pedagogical practice in recent years is the so-called practice of sexting: that is, the “private exchange of self-produced sexual images via cell phone or the internet” (Döring, 2014, 1). Almost no empirical data has been gathered on how sexting is experienced by adolescents in Germany. Additionally, very few studies have been carried out on adolescent experiences of non-consensual image-based sexting in, through and with digital media. For Germany, there is still a lack of reliable data on the prevalence among adolescents (Vogelsang, 2017), evidence on prevalence can be found in United States studies (e.g., the reviews by Döring, 2014; Klettke et al., 2014).

This paper explores the orientations toward the exchange of intimate images and boundary-violating communication through digital media in a school setting. The basis is a qualitative research project, which analyzes the orientations – defined by Bohnsack as patterns of meaning that suggest shared forms of communicable knowledge (2010, 104) – on sexting among school students. The study will address how students interpret and interact with intimate, image-based content, including implications for sexual boundary violations online. Further, the study describes how adolescents position themselves in the field of tension between spaces for possibility and spaces for sexual boundary violation. In the course of the liberalization of society and increasing sexual self-determination in Germany, the former sexual morality has been more and more replaced by a negotiation morality (Schmidt, 1998; Sigusch, 2006). Now the actors – assumed to be equal – themselves define consensus and boundary violation in sexuality in both analog and digital space. The importance of consensus is also reflected in the reform of §177 in the German Criminal Code since 2016, a (sexual) assault is punishable even without any violence or its threat. A special focus will be placed on the reconstruction of orientations toward normalities and sexuality. The study aims to contribute to a more nuanced discourse on both sexting and gendered readings of sexting.

This paper sets out by reviewing recent research on sexual communication and boundary violations through digital media that draws on the phenomenon of sexting and focuses specifically on the aspect of gender (see section “Research on Image-Based Sexual Communication Through Digital Media – Between Self-46 Determination and Boundary Violations”), before detailing the study on which this paper is based (see section “Materials and Methods”). Based on group discussions, see Section “Results” offers insights into the orientation of students in Germany. The paper then summarizes the findings in a conclusion (see section “Discussion”).

## Research on Image-Based Sexual Communication Through Digital Media – Between Self-Determination and Boundary Violations

### Sexuality and Digital Media During Adolescence

Given that sexuality is a socially constituted practice, it is important to consider the specific historical and cultural contexts that inform adolescents’ sexual development. Within this cultural context, sexuality takes shape via highly individual, socially and historically mutable sexual norms and values (Gagnon and Simon, 2000). In the process of sexual socialization, individuals must tackle sexual norms and values; it is through this engagement that they both constitute and contextualize their sexual identities (Marcia, 1980). Drawing on Lanuza (2006) and with reference to Bourdieu’s (2010) terminology, it might thereby be possible to speak of a “sexual habitus,” although this is not yet an established concept in debates that have, to date, focused primarily on psychology.

During the process of sexual socialization (Gagnon and Simon, 2000; Stein-Hilbers, 2000; Simon and Gagnon, 2003), adolescence is influenced by psychosocial and sexual aspects of development (Havighurst, 1972). These could include first intimate and romantic relationships; first explorations of sexual identity; confrontations with (societal) sexual norms, values, and practices; as well as a process of detachment from the parental home (ibid.). All of these aspects are accompanied by increased distancing from adults. It is during the adolescent period, in particular, that individual attitudes, positions, and structures of desire are restructured; pre-existing scripts are updated to realign with an internally perceived, habituated sexual identity (Attwood, 2006). Sexual boundary violations, however, can negatively influence the process of sexual socialization, restricting sexual forms of expression and modes of experience (see for example, Brown et al., 2014).

From the moment the smartphone entered the life of adolescents as an omnipresent device, it has enabled low-threshold, uncomplicated access to the internet and thereby also to sexual communication and interactions online (Hasinoff, 2013). Digital media represent key spaces for information exchange, communication and interaction, which young people use to organize their social and erotic relationships, deal with issues related to sexuality, and engage in (initial) sexual experiences. Online sexual activity is an everyday part of adolescent life. Additionally, Döring (2014) identifies an intensified engagement with media-based sexuality during this period, meaning that young people are increasingly actively turning to various representations and information about sexuality in the media. Through this, they task themselves with independently determining their sex-related media use – and correspondingly, with acquiring the media competency required to do this. This means that drawing a dichotomy between digital and analog sexual communication and interaction is no longer relevant for adolescents today (Kerstens and Stol, 2014; Ringrose and Naezer, 2018). During the transition process between childhood and adulthood, digital spaces offer adolescents countless possibilities to realize their aspirations toward autonomy, testing sexual boundaries beyond parental supervision and control and gaining recognition among their peers (Baumgartner et al., 2015).

A Spanish study on 2,356 high school students documents that being involved in digital sexual communication “does not appear to generate a negative impact among those involved, in a short term.” Adolescents who feel the need to be popular may consider sharing and disseminating sexual content as a strategy to be accepted among their peers (Del Rey et al., 2019, 8). Moreover, the cyberfeminist perspective puts forward the thesis that digital contexts offer new spaces of possibility for the (further) development and differentiation of self-determined and genderqueer sexual identities (Haraway, 1991; Ringrose and Eriksson, 2011). At the same time, digital spaces are also sites where sexual boundary violations can take place (Koops et al., 2018). Because digital spaces harbor specific risks due to their particular contours, insofar as they offer high levels of anonymity, detachment from a concrete time and place, a broad scope, potential high visibility, and the possibility to disseminate content rapidly.

### “Sexting” as an Expression of Intimate Digital Communication

Sexting involves sending intimate sexually explicit messages, photos, or videos via smartphones and computers, and describes a mode of connecting sexuality and digital media (Döring, 2014; Barrense-Dias et al., 2017). As one aspect of digitality experienced by adolescents, it offers an opportunity for a self-determined mode of sexual communication that is generally characterized by reciprocity. By the same token, sexting is a phenomenon that is rapidly changing, meaning that academic studies relating to its definition, but also to its prevalence – as well as researchers’ analyses of it – tend to vary dramatically, and can very quickly become outdated (see Bonilla et al., 2020). The definition of sexting differs depending on the type of media being investigated, as well as – within academic research – on the form (text, photo, and video), the content (ranging from suggestive to explicit in terms of the degree of nudity and the sexual activity depicted), intention (ranging from non-sexual to sexual) and the medium (generally digital forms, from email through to instant messaging). A further factor is the degree to which participants freely engage in sexting (see Döring, 2014; Dekker et al., 2019).

Sexting is often incorrectly equated with cyberbullying, meaning that the dimension of sexuality is rarely taken into account. Nor is sexting a form of “sextortion,” which refers to the attempt to blackmail another person with sexually explicit images for money, for example (Gassó et al., 2019). Additionally, at least in its original definition, sexting does not involve sending or forwarding sexually explicit images without the knowledge and agreement of the represented person or persons – an act that would constitute a criminal offense; nor does it involve recording erotic or intimate images without consent (Strasburger et al., 2019)^1^. Public and academic debates around adolescent sexuality in digital contexts have long revolved primarily around questions of risk. This tendency is most prevalent in debates where the repercussions of adolescent use of pornography are discussed (see for example, Smith et al., 2014). Similarly, in recent years the phenomenon of sexting has increasingly gained attention and been a topic of debate (Dekker et al., 2019). In international publications, sexting is generally classified as a deviant behavior, and accordingly it is discussed from the perspective of the risks it entails (Hasinoff, 2015; García-Gómez, 2019). In particular in United States contexts, sexting is perceived as a risky form of misconduct even when carried out by adults (Döring and Mohseni, 2018; Mori et al., 2019; Wachs et al., 2021). Here, three dangers are assumed: social exclusion and criminal consequences, sexual victimization, and reckless sexual behavior.

While the discourse of deviancy maintains that sexting is an aberrant form of behavior, the normalization discourse emphasizes how widespread digital sexual communication is (Hasinoff, 2013). This discourse frames sexting as a “normal, contemporary form of intimate communication” (Döring, 2015, 25) within a process of sexual socialization that involves a broad segment of adolescents, offering spaces of possibility for the (further) development and differentiation of sexual identities. As Kerstens and Stol state, “research suggests that the Internet provides adolescents with opportunities to explore and express their sexuality” (Kerstens and Stol, 2014, n. page). A meta review by Madigan et al. also concludes the “credence […] that youth sexting may be an emerging, and potentially normal, component of sexual behavior and development.” (Madigan et al., 2018, 332).

In this view, which focuses on the perspective of users, mutually consensual sexting represents a positive and satisfying expansion of one’s own sexual life and relationships, making it an expression of a successful and self-determined sexual identity (Attwood, 2007). Interactions with sexual visual self-representations can therefore be viewed as a significant factor in the culture of adolescent online communication, and a mode of structuring and upholding relationships. This understanding can lead studies to frame sexting as a mutual expression of experimentation with sexual identity, while also addressing the potential for non-consensual exchanges or the extensive dissemination of content without consent, which brings with it psychological and social consequences (see for example, Hasinoff, 2013; Madigan et al., 2018).

Among adults, sexting is common as a form of sexual communication. Data gained from an international meta-analysis of 31 studies show, for example, that more than half of the surveyed adults have sent or received sexts (Klettke et al., 2014). The prevalence of sexting among adolescents has also been the subject of empirical research. A survey study by Madigan et al. (2018) established an average prevalence of 14.8% for sending and 27.4% for receiving sexts among adolescents, although the incidence increases with age, and has been rising in general in recent years. A study in Spain with 3,314 adolescents between the age of 12 and 16 demonstrates that “more than 2 in 25 teenagers send or forward sexual content, while more than 1 in 5 receive it directly from the creator, and more than 1 in 4 teenagers receive it via an intermediary” (Ojeda et al., 2020, 14). According to the meta-analysis of Klettke et al. (2014), in total only 10% of the surveyed adolescents had sent images; 16% indicated that they had received images. Results from a Dutch study with 4,453 adolescent participants “indicated that receiving sexual requests is quite common and that producing sexual images is relatively rare” (Kerstens and Stol, 2014, n. page). In a research project with 357 adolescents, Symons et al. (2018) point out the high probability that sexting will take place in the context of a romantic relationship. A study by Boer et al. (2021) demonstrates different usages of sexting dependent on the gender and socio-economic status of the adolescents.

### Sexting and Sexual Boundary Violations

Sexting becomes the starting point for sexual boundary violations when photos or videos in the correspondence are recorded or forwarded, and in some cases publicly disseminated, without the consent of the persons depicted; or when images are received in an unsolicited manner, that is against the wishes of the recipient. In legal terms, recording and/or disseminating sexually explicit images in a non-consensual manner entails a criminal violation of privacy in most countries. Additionally, such acts, in a German context, violate a person’s right to their own image (Strasburger et al., 2019), as well as constituting a criminal offense in the area of child pornography – although these laws may vary from country to country. Drawing on a Canadian study with 800 adolescents between 16 and 20 years, Johnson et al. (2018) point toward non-consensual sexting as a daily and collectively recognized occurrence, drawing a distinction between the non-consensual forwarding of images via third parties from the consensual sharing of intimate images as an expression of self-determined sexual communication. Boundary violations can negatively influence the process of sexual socialization insofar as self-determined forms of sexual expression and experiences become, through their abuse, restricted (see for example, Brown et al., 2014).

Pre-existing studies that focus on connections between sexual boundary violations and digital media suggest that the manifestations, prevalence, and forms of victimization vary widely in terms of both form and severity. In an older, representative United States study on sexting, 3% of those surveyed indicated that they had forwarded sexually explicit images to third parties without consent at least once (Knowledge Networks, 2009). In a recent study by Barrense-Dias et al. (2020), involving 7,142 adolescents in Switzerland 6% of those surveyed indicated that they had shared sexually explicit photos or videos without consent on one occasion. A further 9% reported that they had done so on multiple occasions. Central motivations included fun (62%), showing off (30%), and a lack of understanding of what they were doing (9%). A study with 4,281portugues adolescents reports of “4.8% engaged in abusive sexting behaviors and 4.3% self-identified as being a non-consensual sexting victim” (Barroso et al., 2021). In the overview study from Madigan et al. (2018), 12% of adolescents surveyed indicated that they had made public a sext without consent, and 8.4% knew that images of their person had been non-consensually disseminated. As Ojeda states, “typically non-consensual sexting behaviors are more frequent than typically consensual ones” (Ojeda et al., 2020, 15).

Overall, it can be assumed that the particularities of digital space (anonymity, scope, speed, visibility, detachment from a concrete time and place) mean that online sexual boundary violations are comparatively more severe than in “analog” spaces (Walrave et al., 2018) – although the relation between the digital and analog experience of boundary violations to date remains unclear. Moreover, isolated cases suggest that challenging situations can be easier to end online (Henry and Powell, 2016). Choi et al., however, demonstrate through a study with 450 adolescent girls with ethnically diverse backgrounds from Texas that “sexting could function as an online extension of offline forms of sexual coercion” (Choi et al., 2016, 167), pointing toward the interconnection between both areas from the perspective of adolescents.

### The Dimension of Gender

Understandings of gender have significant influence on the development of sexual identities, and on the risk of sexual boundary violations. Sexuality and gender identity are developed above all during adolescence. In data on participation in sexting, the non-consensual dissemination of sexually intimate content and the emotional distress this can cause, gender factors are often discussed. In the main, it has been established that there is very little difference in participation levels between boys and girls when it comes to sexting (see Madigan et al., 2018). At the same time, girls and boys are affected by sexting in very different ways (Murray and Crofts, 2015; van Ouytsel et al., 2021). Studies have shown that when the same sexting activities are performed by boys and girls, it is mostly girls who are confronted with negative consequences such as bullying, stigmatization, insults, and slut-shaming when their images are disseminated. While on the one hand women and girls are expected to conform to the hegemonic representation of ideal femininity, on the other hand they run the risk of being labeled “sluts” for suggesting or explicitly showing sexual activity (Naezer and van Oosterhout, 2021, 3). For girls, this can mean that even when they are victims of non-consensual dissemination of their images, they are viewed as being responsible in a typical case of victim-blaming (see for example, Fein, 2011; Ringrose et al., 2013; Bonilla et al., 2020). In non-consensual sharing of intimate images, similar to the dynamics of offline sexual violence, the responsibility for the dissemination of the images is often placed on the victims and not on the publishers and forwarders (Naezer and van Oosterhout, 2021, 4). Boys, on the other hand, tend to be viewed as more masculine through self-generated sexual images (see for example, Ringrose et al., 2013; García-Gómez, 2019). Additionally, boys are more likely than girls to share images without the consent of the depicted person (Morelli et al., 2016a; Johnson et al., 2018; Barrense-Dias et al., 2020). This dynamic reinforces double standards along gendered lines as well as gender inequality (Ringrose et al., 2013). Moreover, it strongly influences both the discourse around sexting, and adolescent orientations toward sexting (Crawford and Popp, 2003; Dobson and Ringrose, 2016).

In this sense, this discussion generally plays out within the dominant order of the gender binary, insofar as the terms “boys” and “girls” create two homogenous groups that reinforce asymmetric vulnerabilities in relation to sexual boundary violations through the representation of gender. A recent Canadian study, however, suggests that the central question is not so much the representation of gender, but the attitudes toward gender stereotypes (Johnson et al., 2018). According to the authors, “youth who believe in traditional gender stereotypes are significantly more likely to share sexts” (ibid., 16). And further: “Although the correlation between adherence to gender stereotypes and sharing behavior is significant for both boys and girls, it is considerably stronger for boys” (ibid.).

### Desiderata

As documented by this brief review of recent research, there are a number of quantitative surveys available on participation in sexting, on non-consensual behavior and the role of gender with regard to sexting. However, Döring (2019, 312) has identified a gap in research on cognitive and emotional processes involved in sexting-related activities, noting that as a result the perspective of adolescents is not taken into account. In particular, there is a need for qualitative studies that reconstruct young people’s orientations in order to understand processes, perceptions and practices (see also Burkett, 2015). Additionally, only rarely is insight gained into how these activities influence sexual biographies, including how the sexual development of adolescents might also profit from self-determined sexual online activity such as cybersex or the consumption of internet pornography (Döring, 2019, 321). However, it is still unclear to what extent digital sexual communication restores stereotypical gender roles and restrictive sexuality norms or, alternatively, enables new spaces of possibility. In this sense, current research points to a desideratum regarding adolescents’ orientations toward sexting as a practice between spaces of possibility and boundary violations.

This paper addresses the ways that adolescent school students deal with intimate digital visual content and offensive communication within digital media. Additionally, it questions how adolescents position themselves in the field of tension between spaces of possibility and sexual boundary violations. A particular focus lies in the reconstruction of orientations toward normalities and gender.

## Materials and Methods

The data gathered by the following empirical reconstruction of school students’ orientations is drawn from the research project ‘SaferSexting – Perspectives of School Students,’ conducted by the BMBF (Federal Ministry of Education and Research in Germany) between 2018 and 2021 within the funding stream ‘Research on sexualized violence against children and adolescents in educational contexts’. The research project looks at sexting and its largely uninvestigated connections to sexuality, non-consensual sexual conduct, digital media, and the school context.

### Sampling

In total, 12 group discussions were carried out in 2018 and 2019 with students aged 16 and 19 at five different secondary schools in rural as well as in urban regions in northern Germany^2^. Two of them were grammar schools (*dt. Gymnasium*) and three (more applied) comprehensive schools (*dt. Gemeinschaftsschule*), so the entire range of the German secondary school system is represented in the sample. Nine of these discussions are taken up in the following analysis. Group discussions with students were gender homogeneous, due to the fact that the current research findings outlined above indicates gendered differences both in relation to sexual boundary violations and sexual communication via digital images. Thus the 12 groups each contained 2 to 6 participants, who were interviewed in a single-sex setting. Five group discussions were held with male participants, seven with female. A total of 42 students (20 boys; 22 girls) took part (see Table 1).

**TABLE 1:** Number of participants and group discussions.

	Boys	Girls	total
Participants (total)	20	22	42
Group discussions (total)	5	7	12

### Data Collection

In order to attract participants, eight schools were asked for participation, that were rated as particularly interesting due to their profile or existing collaborations. The research project was then first discussed with the school administration at five schools and then presented to students aged 16 and 17. The researchers made appointments for the group discussions within the classrooms and during class time with interested students. Each group was composed voluntarily and according to the wishes of the students. The interviews with the girls were carried out by the female research assistant, the group discussions with the boys mostly by trained male members of the research group.

The group discussions were structured by a communicatively validated semi-structured questionnaire that included an initial stimulus on dealing with sexuality and sexual issues in everyday school life to generate narrative (‘‘please tell me: How do you deal with sexuality and sexual issues in your everyday school life?’’) In addition to immanent questions to maintain the narrative exmanent – like ‘‘can you tell more about it’’ or ‘‘do you remember other situations’’ – follow-up questions were asked about sexuality and digital media, sharing of sexts and sexual boundary violation (for example ‘‘what exactly are sexual boundary violations for you’’). The duration of the group discussions ranged between 45 and 106 mins. These were transcribed in accordance with the TiQ (Talk in Qualitative Social Research) guidelines^3^. In total, 17.5 h of material were gathered. The interviews were conducted in German and the interview passages in this text have been translated into English^4^.

### Data Analysis

The analysis of the group discussions draws its methodology from the Documentary Method, in which both implicit and explicit forms of knowledge and discourse are analyzed (Bohnsack et al., 2010). This allows for the identification of collective “orientations” (Bohnsack, 2010, 104), which are assumed to guide actions in everyday social practice based on the notion of “structures of practice” (Bourdieu, 2010). Along these lines, our research in this paper focuses on habituated practices rather than on communicable, explicit knowledge. In the case of group discussions, the task of analysis lies in reconstructing the way discourse is (formally) organized. The group discussions are interpreted, compared and contrasted, before being condensed into identified characteristics or types. In the process of formulating and reflecting on interpretations of the discussions, two predetermined analyzing steps were taken in order to analyze the orientations framing participants’ actions. The construction of types “builds on the components of the framework of orientation common to all the cases” (Bohnsack, 2010, 111). A sociogenic set of types as often prescribed for the documentary method was in this case not possible, as social milieus could not be allocated to the participants on the basis of the two types of school visited; moreover, stable differences in the various orientations prevalent in the discussions vis-à-vis the type of school were not identifiable. Further, the study documents few behavioral differences between genders; rather, differences were present in attitudes held toward gender stereotypes, regardless of the gender of the student. This means that a relational set of types were established that allow specific social formations to be registered, even though their development may not yet be complete or solidified (see Nohl, 2013, 61). This reveals the “systematic context in which the various dimensions of type-specific orientations are found” (ibid., 62).

## Results

The documentary analysis carried out through the relational construction of types yielded a typology with three different orientations: “The Experimenters,” who uncritically view and use sexting as an everyday form of sexual communication; “The Reflexive-Criticals,” who likewise consider sexting to be normal, but are critical of violations; and “The Disapprovers,” who reject all forms of sexual digital communication. Each type contains three of the nine group discussions examined. Group discussions with both girls and boys are represented in each type. In what follows, each type will be described in terms of its orientations toward norms and toward gender, drawing on exemplary excerpts from the group discussions.

### Type A: “The Experimenters”

The first type practices sexting in an experimental fashion. This involves both self-determined and non-consensual forms. The groups *Gamblers* and *Girls’ Night* differ only minimally, in terms of how they position themselves in relation to adolescent ‘normality’ through their actions.

#### Orientations Toward Norms

The students belonging to the group *Gamblers* describe sending sexts as an everyday practice among adolescents that involves both girls and boys.

*B5: It’s kind of like, you hear about it from other people, if someone sends something around, like in the year level or whatever.*
*B3: A dick pic?*
*B5: Yeah or like @(.)@ also of a*
*I: ⌊A what please?*
*B3: A dick pic.*
*B5: Also from the girls’ side. ah you just kind of hear about it and then of course people talk about it, let’s say.*
*[…]*
*B3: Yes. um (1 s) if you have a girlfriend then it’s also, I’d say, pretty normal that you’d get um these sorts of pictures from your girlfriend. and of course ah @you’d then be a gentleman and ah you wouldn’t forward something like that or show it ah to other friends. (1) and (1) I hope after the relationship ends it’d stay that way, that ah it stays anonymous and private, kind of.*
*(Group: Gamblers, 2_S, P:4, 2–11 and 52–58)*

The students frame the practice of sending sexts as being commonplace among fellow students in their year group – a practice that is both widespread and openly acknowledged (the students “hear about it”). Established practices include sending “dick pics” and receiving images “from the girls’ side.” Here, laughter points toward the shared fun of being involved. In particular within heterosexual relationships, for boys, receiving images from their partner is described as being “relatively normal,” and confidentiality in this matter is framed as a question of “honor.” The question of whether sexting takes place with consent is not further discussed by the students, implicitly suggesting that the potential for sexual misconduct or violation here appears to be an irrelevant detail.

Similarly, the orientations of the group *Girls’ Night* are characterized by normative assumptions about sexting as a “normal” sexual form of communication among adolescents.

*G2: Well I would say, because really a lot of people do it, maybe not nudes, but like (.) kind of (.) revealing, ah, images. Sure (.) um on Snapchat. […] and I’d say that I don’t really think it’s bad either, because I think it’s pretty much normal, that you um kind of try it out at some point and also maybe that um you want to get an opinion about yourself, if you’re not really sure. like um if your body or I don’t know it’s like-. that’s a feeling, I think, that lots of girls have, that they need confirmation. so um I don’t mean that in a negative way, that’s I think totally normal, that you um also um just want to know what other people think about you. and that’s why, I think that’s why it’s normal, every girl or also every boy, at some point.*
*(Group: Girls’ Night, P:4, 6–34)*

There is no doubt that the students in this group present sexting for girls as a legitimate space of possibility, of positive affirmation during adolescence. Sexting is a way of attaining validation about one’s appearance and one’s body, and represents a means of dealing with insecurities about one’s own attractiveness. This option is less tied to the recipient of the sext, meaning to exclusively erotic relationships; rather, it represents a legitimate possibility for the sender of the sext to find out “what other people think about you,” on the sender’s own terms. Here, self-recognition and the recognition of others takes place through reciprocal exchange. This process of verification is explicitly framed as being a “normal” adolescent need.

The term “normal” here relate not only to self-determined sexting practices, but also to the non-consensual receipt of sexually explicit images. One female student, for example, recounts having received unsolicited and sexually explicit images. She points out that she believes “plenty of people have received images like that before” – in doing so framing non-consensual sexual communication as a normal activity. The shared laughter in response to her statement documents agreement; the experience of this non-consensual practice is shared by others in the discussion. Speaking through her laughter, the student explains her strategy in dealing with such experiences: “I’m personally not interested in that stuff and I usually just delete the chat or block him or whatever, yeah.” Through this statement, the experience of shame, of having one’s boundaries violated, is not entirely dismissed, but rather is accepted as a part of adolescent experience that can be countered through simple technical steps. Sexual boundary violations are thereby assumed to be a self-explanatory part of sexting, and are accepted as the inevitable negative price to be paid for the personal self-affirmation otherwise afforded by the practice.

#### Orientations Toward Gender

The type “The Experimenters” reflect traditional ideas about constructions of gender, positioning themselves affirmatively within practices that differentiate between two genders. Girls and boys are allocated different roles in the representation of gender, in the sense that boys are sexually active, while girls are positioned on a fine line between the demand to be attractive and sexual passivity. This holds true for the group *Girls’ Night* as well as for the group *Gamblers*, in which this orientation is particularly prevalent.

*B5: Generally though I’d say that the girls cop more than the boys*
*B6: Cop more, what do you mean by that?*
*B3: ⌊What do you mean?*
*B5: ⌊sure, I mean, I know a lot of guys who um would secretly, like, record sex with a girl.*
*B3: ⌊@Oh God@.*
*B5: And uh*
*B6: ⌊Ah, that’s what you mean. yeah, totally.*
*[…]*
*B2: I think it’s often like um, like for example um, […] for guys who don’t post them themselves, but they get shared around a lot anyway um, that it really doesn’t matter, um whether they wanted that or not. but the guy involved, the guy, (.) he isn’t hated on. mostly other guys just say: “Oh, nice one, nice work.” And the girls get hated on. then it’s mostly like*
*B6: ⌊Yeah, like: “Oh, what a slut.” And for the guy: “Oh, what a cool guy.”*
*(Group: Gamblers, P:2, 6–34)*

This orientation points toward gendered differences based on an asymmetric value system involving sexual double standards. In the example detailed, although the boy involved both creates and spreads non-consensual sexual content, he receives confirmation as a “cool guy,” while people develop a “negative impression” of the girl, and she is “hated on” as a “slut.” The boy is completely exonerated of responsibility, while the girl – the victim of the situation – is condemned. In this sense, a judgment is made via a double standard (Döring, 2014; Naezer and van Oosterhout, 2021). The boy is let off the hook, while the girl is denigrated. This orientation is founded in traditional, gendered assumptions on male and female sexuality.

Although the participants appear to be aware of the asymmetries in the effects of these gendered conceptions (as demonstrated by shared agreement), this does not lead them to take a critical position. Participants neither express criticism of the non-consensual recording of sexual content, nor do they address the non-consensual publication of the recordings. Rather, they maintain an apparently neutral narrative voice, meaning that their own position remains ‘suspended’. At the same time, the narrative itself is not at all neutral. Laughter and obvious delight signal a collectively shared agreement that not only supports but amplifies the labeling of the boy involved as a “cool guy,” and the girl as a “slut.” Bindesbøl Holm Johansen et al. argue, that “non-consensual sharing acts as a form of visual gossip to maintain social bonds and gendered recognition” and that this has “gendered implications as it rests on and reproduces gendered values” (Bindesbøl Holm Johansen et al., 2019, 1029). This humorous and ironic approach, working through the narrative, means that normative constructs relating to “doing masculinity” are stabilized (Connell, 2012). Collecting sexts operated as a way “through which boys could gain status and respect among their peers” (Ringrose et al., 2012, 54).

The girls in type A also orient themselves according to gender stereotypes. Along these lines, when the group *Girls’ Night* addresses the non-consensual forwarding of sexually explicit images of girls among boys, they state that “it’s not really that bad when people talk about it.” Thus, practices of sexual misconduct or violation among boys are viewed as “talk” that might be “interesting,” and are thereby marked as being integral for adolescent sexuality. The discrepancy in how boys and girls are affected by these activities is accepted as a given, and is not interrogated.

### Type B: “The Reflexive-Criticals”

The second type is made up of “The Reflexive-Criticals,” represented by two groups of girls and one group of boys. Within type B, two subgroups could be identified: *Negotiators of Responsibility* and *Feminists*. Type B positions itself in opposition to dominant societal notions of normativity and gender (see also Naezer and van Oosterhout, 2021).

#### Orientations Toward Normalities

As with type A, members of the type “The Reflexive-Criticals” indicate active involvement in the practice of exchanging intimate images. However, unlike type A, this orientation is distinguished by a critical distance. The group *Feminists* focuses on perceptions of experiences of boundary violations, leading to a reflection on responsibility and a critique of the contexts in which sexual socialization take place.

*G4: So (.) well mostly on Snapchat and then um he kind of wrote to her and then he like straight away sent her um photos of himself topless and wrote really sexual things to her. I mean really, really exactly like in porn. and um then she didn’t um answer, she goes: “yeah, um leave me alone, I don’t want this stuff.” And then he totally spammed her with photos of his penis, also it was really his penis.*
*G?: ⌊°Shit°.*
*G4: and that and um after that I think she then also blocked him and um you also see stuff like that maybe also sometimes on Instagram*
*G5: ⌊Mhm. ((agreement))*
*G4: And you can also report it. the thing is that Instagram doesn’t usually acknowledge it. No, um not*
*G?: ⌊Nah.*
*G4: n-, really. So I, when I see something like that and report it, because I don’t I find it, I mean, it’s not OK, there are actually pages, they’re like from some people and they um promote child pornography […]*
*(Group: Feminists, P:2.1, 22–40)*

The female students describe an exchange on a social media platform in which an unspecified male individual begins by sending revealing images with “sexual” and “pornographic” texts. The girl refuses this attempt to make contact. Instead of accepting her refusal, the person intensifies the level of boundary violation, “spamming” the girl with images of his penis. Here, too, the female students of the group *Feminists* point toward the option of blocking as a simple technical step to prevent further unwanted contact. Unlike the group *Girls’ Night*, however, the female students here collectively position themselves against an unwanted attempt to initiate contact, including against the sending of images of penises. The group additionally criticize the lack of responsibility of operators of social media services, which react inadequately to reports of sexual misconduct. As the narrative on the topic of social media services continues, they criticize the fact that such platforms do not react when users advertise abusive images of children, so they are confronted with unwanted sexts. Unlike type A, they thereby criticize the described “normalities” as a culture of non-consensual sexual communication experienced by (girl) adolescents, and actively reject it.

In a similar way, the group *Negotiators of Responsibility* discuss a fake account that a fellow student set up on a social media platform. The male student used the account to pretend to be a woman and convince a fellow student to send images of his penis, before showing these images to the class. This practice is rejected by the *Negotiators of Responsibility*.

*B1: Because it just went too far. um, because (.) writing to someone with a fake account and then getting him to um (.) put his um*
*B3: ⌊Private parts.*
*B1: his penis um online. that’s actually- that’s really not cool. that’s really seriously messed up, actually.*
*B3: It’s more than just messed up, it’s*
*B1: ⌊and then to um show this image to others. and laugh about it.*
*B5: Yeah.*
*B1: I didn’t find it funny at all.*
*(Group: Negotiators of Responsibility, P:2, 87–98)*

The students’ strong disagreement with this transgressive practice increases throughout the duration of the conversation in the sense that a boundary is marked out (“it just went too far”). Their shared, normalized judgment of being “really not cool” intensifies to “seriously messed up, actually.” Not only do they condemn the deception of the victim, but they also mark the act of sharing the image within the class – and the resulting ridicule – as an intensification of the boundary violation, and they distance themselves from this act (“I didn’t find it funny at all”).

Type B, then, also see the practice of sexting as a daily component of peer interactions. In other parts of the group discussion, they also detail their own, self-determined experiences with sexting. This type, however, reports above all on the non-consensual sharing and publishing of intimate images, suggesting that a self-determined form of sexual communication online remains an unrealized dream to be fought for. Sexual boundary violations are identified as such, and are rejected without responsibility being relativized. Type B attribute responsibility for violatory sexting practices on a number of levels, but they do not attribute guilt to victims (thereby avoiding both victim-blaming and slut-shaming). This means that they not only discuss the acts of fellow students who perpetrate misconduct and boundary violations, but also address their own behavior, thereby reflecting on possibilities for acting. While boys critically reflect upon their own reactions in retrospect, girls also relate their criticism to further contexts of socialization.

#### Orientations Toward Gender

The type “The Reflexive-Criticals” maintains a critical distance to contemporary gendered sexual norms and taboos (Naezer and van Oosterhout, 2021). The validity of such norms and taboos is rejected by the participants on the basis of their respective orientations. That said, differences are present within type B. While the group *Negotiators of Responsibility* reflect on their own actions, the *Feminists* criticize sexualized power relations and proclaim self-determination.

*G3: (.) But also this thing um with women and how they should present themselves, I just had this big discussion with my sister about it, because she’s taking photos for her Bachelor and um in none of my outfits in the photos was I wearing a bra. and you could see my nipples. and that’s a discussion for sure, in a porno all the guys see naked women and think breasts are super nice. but if a girl walks around with a tight top and isn’t wearing a bra and you can see the breasts and nipples, then it’s all of a sudden this huge drama and I just don’t get it, because it just doesn’t make any sense-, um it makes absolutely no sense to me. These types of things, the way they just*
*[…]*
*G1: ⌊It’s so shitty.*
*G3: ⌊If it was up to me I would just run around on the beach topless.*
*[…]*
*G2: For me it’s like, I don’t wear a bra quite often, and I know that people look at me, but I’m honestly really not interested. they can look if they want. I mean that’s basically their problem. so long as they don’t grope or whatever, I really don’t care what they all think.*
*(Group: Feminists, P:2-2, 1–40)*

The students reflect together on socially accepted norms for clothing for young women. In doing so, they unanimously problematize the restrictive nature of such norms. The three female students recognize the danger of sexualization and of “groping” when young women do not wear a bra under their clothing, or “run around on the beach topless.” The students oppose these restrictive norms with the notion of free choice, protesting that they will wear what they want. They have each had their own experiences of harassment due to their choice of clothing, and take up the position that they “don’t care” what others think of them, “so long as they don’t grope.” In this sense – unlike in type A – a self-determined sexual identity is declared. The threat of violence that sexualization brings with it is also marked as being a part of their experiences. The girls’ self-representations are guided by their own wishes, in contrast to external expectations (from boys/men). The position of being a victim is linked exclusively to the position of girls, and in delineating their own defense strategies, the female students declare their right to self-determination. Boys are referred to in the passage only as “them,” without further detail.

For the male students of this type, resistance to gender norms takes a different form, but it can be compared with the orientations of the female students insofar as it constitutes a break with prevailing notions of gender that legitimize sexual boundary violations. This also becomes apparent through the discussion on the case of the faked account (see section “Orientations Toward Normalities”).

*B5: ⌊Yeah, yeah. (.) Um, for sure um someone from a, from o- our class um made a fake account um on Instagram. and then um sent messages to another guy in our class and pretended to be a woman. and um and sent suggestive messages that she wanted him. um and um the guy from our class, um, he reacted to that. um and I think he then sent um pictures of his penis. (.) the guy who made the fake account, he showed us those images and stuff. and um, so then I told him that I thought what he did was really shitty. because that’s just not OK. and (.) yeah […]. I thought it was totally not OK of him that he pretended to be someone else and (.) um (2) then um lied and stuff. Like, pretended to have feelings for someone else. […]*
*(Group: Negotiators of responsibility, P:2, 12–67)*

As depicted above, the boys indicate that they find the practices detailed to be “really shitty” and “seriously messed up,” because the person “pretended to have feelings for someone” and the images were “shown to others.” In confronting the fellow student in question with their rejection, they take up a position, signal resistance, and take on responsibility in order to clarify the situation. Although – unlike the group *Feminists* – they do not explicitly criticize gender relations, their orientation appears to demonstrate critical distance in relation to the reiteration of forms of masculinity in interactions among males, and refuse to be complicit in hegemonic masculinity (see Connell, 2012). This is demonstrated not only in the confrontation of the perpetrator, as narrated, but also in the rejection of the collective “laughter” in relation to the images. Here, participants revoke the conditioning, through irony and boundary violations, of male dominance, and show responsibility and care, demonstrating an “inclusive masculinity” (Anderson, 2011). The gender-critical potential here lies in a concerted break with the sort of conditioned, hegemonic masculinity that is documented in type A’s orientation.

### Type C: “The Disapprovers”

The third type is identified as “The Disapprovers.” The subgroups *Formalists* and *Values-Oriented Girls’ Group*, who belong to this type, are characterized by the fact that they reject and abstain from digital sexual practices.

#### Orientations Toward Normalities

In the following passage, the students of the *Formalists* group discuss the exchange of sexts as a form of intimate communication. One male student in the group reports of a suggestive conversation with a girl within an otherwise non-romantic friendship, during which the girl offered the boy to send him a sexually explicit picture of herself. The boy is “kind of shocked,” because he had not seen her in this light (“in fact she wasn’t - she wasn’t like that”). This creates occasion for the group *Formalists* to discuss sexting in a largely critical way:

*B1: Yes, it’s because, somehow it’s just assumed to be normal*
*B3: ⌊Yeah.*
*B1: That’s my feeling. I don’t know. or people don’t think it’s so bad or something.*
*B3: Yeah, that’s it, yes, that’s how it is. and it’s your business if you send something like that or if you do something like that. that’s always what (.) people say in the end.*
*B1: Mh.*
*B3: But for me, I have to say I’m glad, um, that I’ve never received something like that or anything else, because I, (.) I’m just fundamentally against sending things like that at all.*
*(Group: Formalists, 4-S_2, P:6, 61–73)*

The students criticize the assumption that image-based intimate communication is normal. In doing so, they suggest that the consensual exchange of images should also be refused. They are guided discursively by restrictive moralizing norms, and reject an orientation toward consensual processes of negotiation and individual possibilities for action.

The *Values-Oriented Girls’ Group* also distances itself from the practice of exchanging images. In the following passage, the students discuss a situation in which the sext of a fellow student is made public among their year group. The event is associated, for these students, with “forgetting.”

*G1: So I-. so, if we’re talking about images-. So, I can still remember. I don’t know if you know, um. […] but, a girl from our year group sent her boyfriend a shot of her arse and that image also got around.*
*G2: Ah, yeah.*
*G4: Yes.*
*G3: Yes.*
*I: And what happened?*
*G1: So the girl said it wasn’t her in the picture, although you could te-tell that it was her jumper and stuff in the picture. and yeah, then people shit-talk about her. a- like calling her bitch, whore and stuff. And yeah.*
*G4: But also with things like that for example. so I wouldn’t really have thought about it again. things like that also get forgotten pretty quickly I think.*
*G2: Yeah.*
*[…]*
*I: Can you say a bit more about it, what it was like and what happened afterward?*
*G1: So I don’t know exactly really. but, the girl sent it to her boyfriend. and when they broke up, the image like, (.) the image got around. and then some people got in trouble for sending it and stuff, actually in my friend group. um, and (.) yeah, then there was a fight. then there were arguments too, yeah, you’re not my friend anymore, that’s, (2)*
*G4: ⌊Mhm*
*G1: ⌊yes so, but (.) we were younger back then. that all happened one, maybe 2 years ago. and yeah, (.) now i- it’s more or less forgotten. now people don’t really think ab- about it and it’s forgotten. (2) Yeah.*
*(Group: Values-Oriented Girls’Group, 4-S_innen_2, P:2, 1–58)*

The group reports that an intimate image was forwarded – although the formulation “the picture got around” side-steps naming an involved human actor in this process. The female student depicted in the image was apparently “shit-talked” as a “bitch, whore.” The denigration of the girl, including using an insult that implies she might be selling sex, is not contradicted by the participants in the group discussion. Instead, the shared act of remembering is coupled with the statement that “things like that … get forgotten pretty quickly.” That the image was shared among their immediate friends is a fact, yet the *Values-Oriented Girls’ Group* positions itself as being detached from the events. They are only affected in the sense that there were arguments that arose within their own friend group; the girl who is the victim of the incident is not mentioned at all. Thus it is not the event itself, but rather its victim that has been forgotten. Questions relating to the responsibility of the person who shared the image, or about caring for a fellow female student whose rights were violated, are not important to the *Values-Oriented Girls’ Group* – unlike for type B. The only relevant frame of reference is one’s own circle of friends, which in this case was threatened with division due to an argument over possible participation in sharing the image. By using “forgetting” as a model for defense and repression, the *Values-Oriented Girls’ Group* positions itself as being beyond the practice of sexting and denies its own involvement in this practice.

#### Orientations Toward Gender

Those belonging to the type “The Disapprovers” position themselves as being both outside of, and individually unaffected by, societal discourses in relation to gender. Although this type refers to pre-existing gender differences, and even reflects on them by drawing on examples of gender-specific clothing, they dismiss these differences as being irrelevant on a personal level.

*G4: Ah yes, um, there’s also a boy, wh-who I also know. and his girlfriend also like online (.) very provocatively-. I mean, they broke up. and she reacted to that very provocatively […] I mean, writing that he’s a son of a bitch, that sort of thing. and that you-, that we should keep away from that wanker and that kind of thing. and, um, so that was then-. that was what happened. and this girl for example, she’s pretty explicit. now she shares around that she has a new boyfriend, and she shares things about herself around a lot too. I mean, like she’s in a white shirt, only has a bra and undies on, and her boyfriend’s lifting her up, that kind of thing. I mean, she also uploads stuff like that.*
*(Group: Values-Oriented Girls’ Group, P:4, 151–162)*

This female student from the *Values-Oriented Girls’ Group* details the behavior of another girl who sends out “very provocative” digital warnings after the end of her relationship. The students criticize that the girl publishes intimate images of herself with her new boyfriend. Similar to type A, here, a denigration of feminine erotic self-representations takes place in the mode of slut-shaming, without further connecting this to gender roles. Males are mentioned in this narrative only as ‘appendages’ of the apparently ‘morally corrupt’ actions of the girl. The representation focuses exclusively on the individual orientations of the girl; the narrative voice remains consistently distanced. A personal criticism is not formulated, nor are alternative forms of sexual communication mentioned. Rather, the behavior is detailed from a distanced position.

The boys’ group the *Formalists*, too, entirely exclude gender relations from their narrative, and do not reflect on the framework of social conditions. Rather, the orientation they bring to the conversation is an individualization that eradicates gendered difference. For example, they state that it does not make a difference if girls are seen in the changing room in their underwear or in a bikini in the swimming pool. Additionally, they do not recognize a difference between boys and girls in underwear, establishing that there is no need to make “a big drama” out of such aspects. Through this – unlike with type B – prevailing gender relations are not criticized, but are denied completely and in that sense also implicitly fortified.

## Discussion

This research project investigates sexting among adolescents through the interrelation between sexual boundary violations and digital media, aspects that have rarely been illuminated to date. As described above, adolescence is a key phase in the process of sexual socialization – a phase that increasingly unfolds in part via digital media. Digital media, like analog spaces, provide both spheres of possibility for the crystallization of a self-determined sexual habitus, and also pose risks due to the possibility of non-consensual sexual communication. Through such boundary violations, the process of sexual socialization can be negatively influenced, insofar as it restricts self-determined sexual forms of expression and modes of experience (see, for example, Brown et al., 2014). According to the most recent research findings, girls are more affected by this than boys. This clear point of departure opens out toward gaps in recent research, in particular in relation to the orientations of adolescents. The group discussions were analyzed with this in mind, in order to identify which orientations adolescent students exhibit in relation to their modes of dealing with intimate digital images, as well as with boundary-violating communication taking place through digital media.

## Conclusion

Working with the method of forming relational types, this analysis allows three different types to be identified:

For type A, “The Experimenters,” sexting is viewed as an everyday part of intimate communication among adolescents, and one which offers a space of possibility for recognition in the eyes of others. Sexting is thereby understood as a space where individually determined possibilities might unfold. This can also imply risks, as in this view, boundary violations are assumed to be part and parcel of sexting practices in the process of structuring sexual communication on a daily basis. The responsibility for boundary violations is identified as lying in the hands of the producers of the images themselves. The validation students receive and the spaces of possibility these activities offer, as well as the risks involved, are understood as being normative in the process of adolescent experimentation with sexuality. Those who are not familiar with how to protect themselves (technically) are ‘themselves to blame’: this is the individualizing assumption operating within this orientation. Such a construction of normativity goes hand-in-hand with orientations toward gender stereotypes that allow sexually active boys to fulfill masculine norms with confidence and irony, objectifying masculinity (García-Gómez, 2019). On the other hand, sexually active girls are understood to be responsible when images are shared in a non-consensual manner, and are confronted with victim-blaming and slut-shaming (Attwood, 2007). The asymmetrical modes of judgment at play when boys and girls are subjected to boundary violations are acknowledged by adolescents, but they are seen as being a normative part of adolescent reality. Here, traditional gender stereotypes dominate (Ringrose et al., 2013).

Members of type B, “The Reflexive-Criticals,” are also involved in sexting practices. While for type A, adolescent experimentation and the possibility for mutual recognition are foregrounded, type B additionally reflects on the non-consensual forwarding and publishing of intimate images as a transgressive practice. In this group, such practices are repudiated. The normativity of adolescent affirmation through sexting is acknowledged and – in the spirit of sexual self-determination – both actively claimed and partly tested out, even as it is called into question as a potentially precarious illusion due to boundary violations. The efficiency of digital reality is framed as being inimical to the potential of self-defined spaces of possibility. Type B does not place responsibility for non-consensual sexual content in the hands of the persons depicted, but seeks to identify those responsible, while posing questions about personal possibilities for effecting change. “The Reflexive-Criticals” thereby distance themselves from societal gender stereotypes (Anderson, 2011). Through their actions, the boys contradict traditional notions of masculinity which do not respect the personal boundaries of women: they confronted the perpetrator and reflected on possibilities for supporting the victim (although these measures were not taken). The girls of this type, on the other hand, criticize normative and restrictive demands of femininity that lead to boundary-violating behavior, which they see as curtailing their desire to realize sexual self-determination (Dobson and Ringrose, 2016).

Type C, “The Disapprovers,” distances itself from digital adolescent cultures that engage in sexual forms of communication and performances of gender. Students belonging to this type view sexting in general as a practice carried out by ‘others’, a practice they say they do not come into contact with. In their orientations, they view sexting in a blanket way as a “non-normative” practice. Its potential significance for adolescent culture is refuted, and – unlike for type A and B – there is no identifiable interest in adolescent experimentation through intimate digital communication. The relevance of sexual digital communication for one’s own sexual socialization is rebuffed. This abstinent orientation is applied not only to these student’s own positions, but is extended to others who view sexting as a legitimate part of sexual communication. In line with this wholesale rejection, the orientations of type C do not differentiate between consensual and non-consensual sexting practices. Rather, they consider being confronted with sexual communication as a boundary violation in itself. Orientations toward gender, too, renounce differentiation in favor of a supposed existence of equality. Gender-based asymmetries in the experience of sexting are not reflected upon, meaning that gendered stereotypes and power imbalances are implicitly reproduced. This presumed neutrality indicates that orientations to sexual norms that are understood as different are excluded and marginalized. The distanced position in relation to sexting practices makes it impossible to recognize non-consensual sexting practices, meaning that responsibility for boundary violations is *de facto* ascribed to the depicted person. The experimentation of autonomy of this type in the course of sexual socialization occurs in the form of boundary violations against persons considered not being male.

**FIGURE 1:**
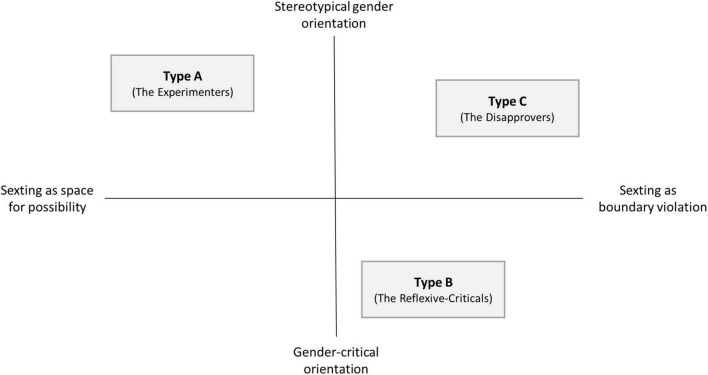
Types and orientations.

The interviewed adolescents position themselves within the field of tension between spaces of possibility and boundary violations (see Figure 1). Most of the students consider sexting to be a risky practice because of the potential for sexual boundary violations; only one type shows normality in the use of sexting. Thus, the study confirms the reported findings that understand sexting as a normal part of adolescent sexual communication, however, this practice is by no means commonplace among the adolescents interviewed (Döring, 2015; Madigan et al., 2018). While many young people are familiar with sexting practices and are involved in various ways, this does not mean that they actively use sexting themselves. At the same time, some of the young people are interested in experimenting with image-based intimate digital communication in the process of sexual socialization and would like safe spaces for this, where they can practice consent and get help from adults if something unpleasant happens to them in their dealings with intimate content. It becomes clear that only type A experiences sexting as an unrestricted field of possibility; in doing so, this type aligns itself with the normalization discourse around sexting. In this context – in which their sexual identity finds a space to crystallize –, those belonging to this type seek and experience recognition through sexting, but also receive sexual material non-consensually through this practice. Type A reflect upon their orientations toward norms primarily in relation to themselves. This means that boundary violations are seen as being normal; beyond deploying technical measures (“blocking”), they are not further problematized. Type B, on the other hand, imagines sexting to represent a possible self-determined mode of sexual communication that, due to existing patterns of behavior, is considered to be precarious and risky. The wish to realize a self-determined sexual identity is constantly threatened by societal norms relating to sex and gender, and the normalization of hierarchical gender stereotypes. Type B, however, also sees the possibility for action when boundary violations take places as being a matter of their own initiative without support from teachers or other actors in school. In comparison to Type A and Type C, for this type social recognition is less dependent on external factors but more influenced by the struggle for self-recognition. On the other hand, when boys and girls of this type come together as a group, they show greater empathy toward victims of sexual boundary violations in their social environment than do those from the other two types. On the opposite, type C aligns itself with the deviancy discourse, rejecting sexting as a form of intimate communication and characterizing it as fundamentally threatening and abnormal. This type, like type A, relates its normative orientations toward sexting primarily to individual experiences. The crystallization of sexual identity here is viewed as taking place in opposition to intimate digital communication, while the normativity of gender stereotypes and boundary violations is not questioned. Overall, for the majority of adolescents, sexting does not offer a space of possibility for a self-determined sexual identity.

Gender-critical positions are present only within type B. Gender stereotypes are explicitly reproduced by type A, and implicitly by type C. This finding is particularly relevant in light of the tendency to slut-shame when dealing with sexually experimental girls, and to victim-blame in order to ascribe responsibility for boundary violations to (for the most part female) victims, as also reported in other studies (Fein, 2011; Ringrose et al., 2012, 2013; Bonilla et al., 2020; Naezer and van Oosterhout, 2021). These patterns form a central orientation toward the dimension of gender, both for “The Disapprovers” and “The Experimenters.” Type B, however, criticizes these patterns; members of this type do not seek to ascribe responsibility to the victim. Given that in all three types both boys and girls are represented, these orientations are evidently not dependent on the category of gender. Far more, victim-blaming and slut-shaming are closely linked to shared orientations toward gender (Johnson et al., 2018; Bindesbøl Holm Johansen et al., 2019). In this way, orientations toward gender stereotypes ‘favor’ both the attribution of responsibility for boundary violations to girls (and in isolated cases also to boys who publish images), and overlook the responsibility of the boys and girls who perpetrated the boundary violation in the first place. One should not mistakenly equate the normality shown by young people during group discussions with a consensual attitude. Rather, adolescents of all types find ways to deal with the fact that boundary violations are part of normal sexual socialization. By implication, the adolescent’s characteristic striving toward autonomy during sexual socialization involves a normalization of boundary violations However, as the expression of a collectively shared orientation that transcends gender and generation, this also limits young people’s ability to address boundary violations vis-à-vis their peers or adults.

Beyond this, the general construction of normativity is linked to orientations toward gender. Those types who view sexting exclusively within the horizon of individual orientations toward norms – regardless of whether they support or reject the practice – tend to reinforce gender stereotypes and negate real boundary violations, in particular through the mode of slut-shaming (on the significance of gender roles, see also Morelli et al., 2016a,b). Thus, orientations toward gender stereotypes, and the acceptance of non-consensual sexual communication as a normality, are mutually co-dependent on one another.

### Limitations

Limitations of the study exist due to the sex-homogeneous design of the group discussions, which may contribute to reifications of gender. In addition, non-binary youth are not represented in the sample. Further, only few adolescents participated in the study who reported own positive experience with intimate digital communication. A broader study with a larger number of participants with diverse backgrounds could differentiate and validate the findings. Including non-binary youth, as well as broader consideration of diversely oriented youth overall, would help to further differentiate the findings on dichotomous heteronormative understandings of normality. It could also lead to greater sensitivity to the risk of reifying these adolescents through the research process. Furthermore, in order to explicitly reach non-binary and gender-non-conforming youth and fruitfully explore their lifeworld interaction via digital media and sexuality, we would need a more gender-diverse sampling. In addition, lgbq adolescents would have to be explicitly addressed, since a significantly higher level of media-mediated erotic and sexual communication is recognizable in this group (Beyenns and Eggermont, 2014).

### Outlook

Further studies would need to look for settings in which a positive attitude toward sexting is associated with a gender-critical orientation. Further research is needed with mixed-sexed groups to control for possible gender bias. However, a study based on mixed-gender group discussions of adolescents in Australia generated findings similar to our analysis (Albury, 2015). In addition, it would be important to analyze the orientations of non-binary adolescents. Furthermore, studies on the effects of pedagogical interventions in cases of sexual violation would be necessary. Because as a practical result, apparent attempts to prevent boundary violations – which primarily address the responsibility of girls – not only encourage the tendency toward victim-blaming, but reinforce both gender-stereotypical orientations and normalizations that tend to restrict the formation of self-determined sexual identities.

## Data Availability Statement

The raw data supporting the conclusions of this article will be made available by the authors, without undue reservation.

## Ethics Statement

The studies involving human participants were reviewed and approved by the Ethics Board of the German Educational Research Association. The participants provided written informed consent to participate in this study.

## Author Contributions

JB initiated the study and did the sections Introduction, Methodologies, and Findings. JB and CW collaborated the data preparation and analysis, did the section Results, and wrote the literature review section. All authors discussed the design, interpreted the results, contributed to drafting and revision of the manuscript, and approved the final manuscript.

## Conflict of Interest

The authors declare that the research was conducted in the absence of any commercial or financial relationships that could be construed as a potential conflict of interest.

## Publisher’s Note

All claims expressed in this article are solely those of the authors and do not necessarily represent those of their affiliated organizations, or those of the publisher, the editors and the reviewers. Any product that may be evaluated in this article, or claim that may be made by its manufacturer, is not guaranteed or endorsed by the publisher.

## Footnotes

1 Relevant here is the fact that, according to German law (§184c, [4]), the self-determined exchange of intimate images by adolescents does not constitute child pornography insofar as those involved have created the images exclusively for personal use and with the consent of the person(s) depicted (Hoven, 2018).

2 It proved to be more difficult to find boys who were willing to participate in group discussions than girls, it was also more difficult to bring together interested student groups in (more academic) grammar schools (*Gymnasien*) than in (more applied) comprehensive schools (*Gemeinschaftsschulen*) in the multi-tiered German school system.

3 The TiQ guidelines as outlined in Bohnsack et al. (2010) implement a number of notational shorthands, including: *(.)* Pause up to one second; *(2)* Number of seconds of a pause in speech;⌊ overlapping speech acts; *hello-* termination of a word; *combi:ned* elongation; *@no@* laughing, *@.@* short laughter,°*Oh*° spoken very quietly. In this instance, the interviewer is abbreviated to *I* and interview subjects are allocated with a number and the prefix *G* (girl) or *B* (boy).

4 The quality of the translation was controlled by external experts.
